# Novel CS1 CAR-T Cells and Bispecific CS1-BCMA CAR-T Cells Effectively Target Multiple Myeloma

**DOI:** 10.3390/biomedicines9101422

**Published:** 2021-10-09

**Authors:** Vita Golubovskaya, Hua Zhou, Feng Li, Robert Berahovich, Jinying Sun, Michael Valentine, Shirley Xu, Hizkia Harto, John Sienkiewicz, Yanwei Huang, Lijun Wu

**Affiliations:** 1 Promab Biotechnologies, 2600 Hilltop Drive, Richmond, CA 94806, USA; hua.zhou@promab.com (H.Z.); feng.li@promab.com (F.L.); robert.berahovich@promab.com (R.B.); sunnie.sun@promab.com (J.S.); michael.valentine@promab.com (M.V.); shirley.xu@promab.com (S.X.); hizkia.harto@promab.com (H.H.); john.sienkiewicz@promab.com (J.S.); yanwei.huang@promab.com (Y.H.); 2 Biology and Environmental Science College, Hunan University of Arts and Science, Changde 415000, China; 3 Forevertek Biotechnology, Janshan Road, Changsha Hi-Tech Industrial Development Zone, Changsha 410205, China

**Keywords:** chimeric antigen receptor, CAR-T cells, CS1, BCMA, immunotherapy, cell therapy, tumor antigen, multiple myeloma

## Abstract

Multiple myeloma (MM) is a hematological cancer caused by abnormal proliferation of plasma cells in the bone marrow, and novel types of treatment are needed for this deadly disease. In this study, we aimed to develop novel CS1 CAR-T cells and bispecific CS1-BCMA CAR-T cells to specifically target multiple myeloma. We generated a new CS1 (CD319, SLAM-7) antibody, clone (7A8D5), which specifically recognized the CS1 antigen, and we applied it for the generation of CS1-CAR. CS1-CAR-T cells caused specific killing of CHO-CS1 target cells with secretion of IFN-gamma and targeted multiple myeloma cells. In addition, bispecific CS1-BCMA-41BB-CD3 CAR-T cells effectively killed CHO-CS1 and CHO-BCMA target cells, killed CS1/BCMA-positive multiple myeloma cells, and secreted IFN-gamma. Moreover, CS1-CAR-T cells and bispecific CS1-BCMA CAR-T cells effectively blocked MM1S multiple myeloma tumor growth in vivo. These data for the first time demonstrate that novel CS1 and bispecific CS1-BCMA-CAR-T cells are effective in targeting MM cells and provide a basis for future clinical trials.

## 1. Introduction

Cellular immunotherapy is an emerging and highly promising approach for the treatment of cancer. T cells constantly sample MHC-presented peptides for foreign antigens and can discriminate abnormal (cancer or infected cells) from normal cells [1,2,3,4]. Genetically modifying T cells with CAR (chimeric antigen receptor) constructs is the most common approach to generate tumor-specific T cells [5,6,7,8] and does not require consideration of patient HLA type. CAR-T cells targeting tumor-associated antigens (TAA) can be infused into patients (called adoptive cell transfer or ACT), representing an efficient immunotherapy approach [9,10,11,12,13,14].

CS1 (SLAM family member 7, CD319) and BCMA (tumor necrosis factor receptor superfamily member 17) proteins are often overexpressed in multiple myeloma [15,16,17,18,19]. Based on their high percentage of expression in multiple myeloma, both targets are used for CAR-T cell therapy [19,20,21,22]. One of the challenges is that BCMA can be downregulated or lost, causing resistance to this treatment; thus, novel CAR-T cells and bi-specific CAR-T cells need to be developed for effective therapy of multiple myeloma similarly to bispecific CD19–CD22, CD19–CD20, CD19–CD37, and other state-of-the art CAR-T cells developed against leukemia [23,24,25,26,27,28]. This report aimed to develop novel CS1-CAR-T cells and bispecific CS1-BCMA-CAR-T cells against multiple myeloma.

In this study, we isolated a new monoclonal CS1 antibody (clone 7A8D5) that binds the CS1 antigen and used it for generation of CS1-CAR-T cells. The novel CS1 (7A8D5 ScFV)-CAR-T cells killed CHO-CS1 cells and multiple myeloma and secreted IFN-gamma. Next, we designed bispecific CS1 (7A8D5 scFv)-BCMA (4C8A clone Scfv [29])-41BB-CAR-T cells that targeted both CHO-CS1 and CHO-BCMA cells and MM cell lines positive for CS1 and BCMA. In addition, novel CS1 and CS1-BCMA-CAR-T cells effectively blocked MM xenograft tumor growth in vivo. This report, for the first time, demonstrates high efficacy of novel CS1-CAR-T cells and CS1-BCMA CAR-T cells in targeting multiple myeloma and provides a basis for future clinical studies.

## 2. Materials and Methods

### 2.1. Cell Lines

Raji, RPMI8226, MM1S, K562, and CHO cell lines were purchased from the ATCC (Manassas, VA, USA) and cultured either in DMEM (GE Healthcare, Chicago, IL, USA) or RPMI-1640 medium (Thermo Fisher, Waltham, MA, USA) containing 10% FBS (AmCell, Mountain View, CA, USA). CHO-CS1 cells were purchased from BPS Bioscience (San Diego, CA, USA) and cultured in Ham’s F12K medium containing 10% FBS and 1 mg/mL geneticin Thermo Fisher (Waltham, MA, USA). Hela-CS1 cells were generated from HeLa cells by transducing with CS1 cDNA lentivirus. Human peripheral blood mononuclear cells (PBMCs) were isolated from whole blood obtained in the Stanford Hospital Blood Center, Stanford according to IRB-approved protocol (#13942). PBMCs were isolated by density sedimentation over Ficoll–Paque (GE Healthcare) and cryopreserved for later use.

### 2.2. Antibodies and Recombinant Proteins

CS1 (CD319) mouse monoclonal antibody (clone 7A8D5) was purchased from Promab (Cat#32123). Control monoclonal CS1 antibody was purchased from Biolegend, (San Diego, CA, USA) (clone 162.1 Cat#331806). Recombinant proteins CS1 protein and PINK recombinant protein were obtained from Promab. For ELISA assay, HRP labeled anti-Mouse IgG was used from Sigma (Cat#: A0168). CD3, CD8, CD4, CD45RA, and CD62L conjugated antibodies and 7AAD were all purchased from Biolegend (San Diego, CA, US). Human serum and goat anti-mouse F(ab)’2 or anti-human F(ab)’2, antibodies were purchased from Jackson Immunoresearch (West Grove, PA, USA). Streptavidin PE was purchased from BD.

### 2.3. CS1 Antibody and Clone 7A8D5 Generation

Six to eight week old BALB/c mice were immunized by subcutaneous injection. with the recombinant fusion CS1 protein extracellular domain (23–226 amino acids). For hybridoma generation, the immunized mouse splenocytes were fused with SP/0 myeloma cells using PEG (polyethylene glycol) and HAT (hypoxantine–aminopterin–thymidine) medium selection. Hybridomas were diluted using 96-well plates to obtain single clones and screened by ELISA for selection of positive clones using the immunogen. The positive hybridoma clones were cultured and expanded to produce anti-CS1 antibodies. The supernatants of these antibody clones were collected, purified using Protein G column, and analyzed by Western blotting and FACS. The best positive clone 7A8D5 was selected and used for VH and VL sequencing for CAR generation.

### 2.4. Lentiviral CAR Construct

The codon-optimized sequence CS1 (7A8D5) ScFv was synthesized in IDT as a Gblock and subcloned into a second-generation CAR sequence with either CD28 costimulatory domain or 4-1BB costimulatory domain and CD3 activation domain. For CS1-BCMA bispecific CAR, BCMA scFv (clone 4C8A) [29] was used with a 41BB costimulatory domain and CD3 activation domain. The CAR was in subcloned into a third-generation lentivirus under either EF1 (with CD28 costimulatory domain CAR) or MNDU3 promoter (with 41BB costimulatory domain CAR). Mock CAR-T cells with extracellular TF tag-CD28-CD3 CAR-T cells were used as Mock CAR-T cells [24,30].

### 2.5. Lentivirus Generation

A total of 2.5 × 10^7^ HEK293FT cells were seeded on 0.01% gelatin-coated 15 cm plates and cultured overnight in DMEM, 2% FBS, 1× pen/strep. The cells were transfected with 10 µg of the CAR lentiviral vector and the pPACKH1 Lentivector Packaging mix (System Biosciences, Palo Alto, CA, USA) using the NanoFect transfection NF100 agent (Alstem, Richmond, CA, USA). The next day, the medium was replaced with fresh medium, and, after 48 h, the medium with lentiviral particles was collected. The medium was cleared of cell debris by centrifugation at 2100× *g* for 30 min. The virus particles were concentrated by ultracentrifugation at 112,000× *g* for 60 min at 4 °C using an SW28.1 rotor, resuspended in serum-free DMEM medium, and frozen in several aliquot vials at −80 °C.

### 2.6. CAR-T Cells

PBMC were suspended at 1 × 10^6^ cells/mL in AIM V-AlbuMAX medium (Thermo Fisher, (Waltham, MA, USA) containing 10% FBS and 10 ng/mL IL-2 (Thermo Fisher, Waltham, MA, USA)) and activated by mixing with an equal number of CD3/CD28 Dynabeads (*Thermo Fisher,* Waltham, MA, USA) in nontreated 24-well plates (0.5 mL per well). At 24 and 48 h, lentivirus was added to the cultures at a multiplicity of infection (MOI) of 5–10. The T and CAR-T cells proliferated over 10–12 days with medium changed every 3 days to maintain the cell density at 1–2 × 10^6^ cells/mL.

### 2.7. Flow Cytometry (FACS)

First, 0.25 million cells were suspended in 100 µL of buffer (PBS containing 2 mM EDTA pH 8 and 0.5% BSA) and incubated on ice with 1 µL of human serum for 10 min. The diluted primary antibody was used with cells for 30 min at 4 °C, and then, after washing, the biotin-conjugated goat anti-mouse F(ab)’2 was added with CD3-APC-conjugated mouse α-human CD3 antibody and PE-conjugated streptavidin at 1:100 dilution, before incubating for 30 min at 4 °C. The cells were rinsed with 3 mL of washing buffer, then stained for 10 min with 7-AAD, suspended in the FACS buffer, and analyzed on a FACSCalibur (BD Biosciences, San Jose, CA, USA). Cells were gated first for light scatter versus 7-AAD staining, and then the 7-AAD live gated cells were plotted for anti-CD3 staining versus CAR-positive staining with anti-(Fab)2 antibodies.

### 2.8. Immunohistochemistry (IHC)

Normal and tumor tissue sections (4 µm) were deparaffinized in xylene twice for 10 min, then hydrated in graded alcohols, and rinsed in PBS. Antigen retrieval was performed for 20 min using 10 mM citrate buffer, pH 6.0. The sections were cooled, rinsed with 1× PBS and incubated in a 3% H_2_O_2_ solution for 10 min. For blocking, the tissue sections were incubated in goat serum for 20 min and then incubated with primary CS1 antibody. Then, sections were incubated with biotin-conjugated goat anti-mouse IgG for 10 min, rinsed with PBS, incubated with streptavidin-conjugated peroxidase for 10 min, and rinsed with PBS. Finally, the sections were incubated in DAB substrate solution for 2–5 min, counterstained with hematoxylin, rinsed with water, and dehydrated in graded alcohols and xylenes. Coverslips were mounted with glycerin. Images were acquired on a Motic DMB5-2231PL microscope with Images Plus 2.0 software.

### 2.9. Cytotoxicity (Real-Time Cytotoxicity Assay)

Adherent target cells (CHO-CS1; CHO; Hela-CS1 or Hela) (1 × 10^4^ cells per well) were seeded into 96-well E-plates (Acea Biosciences, San Diego, CA, USA) using the impedance-based real-time cell analysis (RTCA) × CELLigence system (Acea Biosciences, San Diego, CA, USA). The next day, the medium was removed and replaced with AIM V-AlbuMAX medium containing 10% FBS ± 1 × 10^5^ effector cells in triplicate (CAR-T cells or non-transduced T cells). The cells were monitored for another 24–48 h with the RTCA system, and impedance was plotted over time. Cytolysis was calculated as (impedance of target cells without effector cells minus impedance of target cells with effector cells) × 100/impedance of target cells without effector cells.

### 2.10. IFN-Gamma Secretion Assay

Nonadherent target cells (Raji, MM1S, K562) were cultured with the effector cells (CAR-T cells or non-transduced T cells) at a 1:1 ratio (1 × 10^4^ cells each) in U-bottom 96-well plates with 200 µL of AIM V-AlbuMAX medium containing 10% FBS, in triplicate. After 16 h, the top 150 µL of medium was transferred to V-bottom 96-well plates and centrifuged at 300× *g* for 5 min to pellet any residual cells. The top 120 µL of supernatant was transferred to a new 96-well plate and analyzed by ELISA for human IFN-γ levels using a kit from R&D Systems (Minneapolis, MN, USA) according to the manufacturer’s protocol. The supernatant after RTCA with adherent target cells was collected and analyzed as above.

### 2.11. CAR-T Cell Expansion in G-Rex System

To expand CS1-BCMA CAR-T cells, 10 million cryopreserved PBMCs were activated with anti-CD3-anti-CD28 beads in 10 mL of AIM-V medium (Thermo Fisher, Waltham, MA, USA) containing 10 ng/mL human IL-2. The medium was supplemented with 5% Immune Cell Serum Replacement (Thermo Fisher, Waltham, MA, USA) instead of 10% fetal bovine serum (FBS). The next day, CS1-BCMA CAR lentivirus was added to the cells along with a transduction enhancer. Virus was also added at 48 h after T-cell activation. On day 4, the CAR-T cells were transferred to a 1 L G-Rex container (Wilson Wolf, Saint Paul, MN, USA) along with 900 mL of fresh medium. On day 10, the medium was removed, the cells were suspended in the residual medium, and the cell density was determined using a hematocytometer with trypan blue. The manufactured CAR-T cells were tested for expansion and functional tests in fresh and frozen samples.

### 2.12. NSG Mouse Tumor Xenograft Model and Imaging

Six week old male NSG mice (Jackson Laboratories, Bar Harbor, ME, USA) were housed in accordance with the Institutional Animal Care and Use Committee (IACUC) (#LUM-001). Each mouse was injected subcutaneously on day 0 with 100 µL of 1.5 × 10^6^ MM1S-luciferase positive cells in sterile serum-free medium. On the next day, 1 × 10^7^ CAR-T cells in serum-free medium were injected intravenously. Imaging was done after luciferin injection using Xenogen Ivis System (Perkin Elmer, Waltham, MA, USA). Quantification was done by measuring bioluminescence (BLI) in photons/sec signals. The Kaplan–Meier survival curve was plotted with GraphPad Prism software using mouse survival data.

### 2.13. Statistical Analysis

Data were analyzed with Prism software (GraphPad, San Diego, CA, USA). Comparisons between two groups were performed by unpaired Student’s *t*-test; comparisons between multiple groups were done with one or two-way ANOVA followed by Sidak or Dunnett’s tests. A *p*-value <0.05 was considered significant.

## 3. Results

### 3.1. CS1 Antibody Clone 7A8D5 Binds CS1 Protein

**Figure 1:**
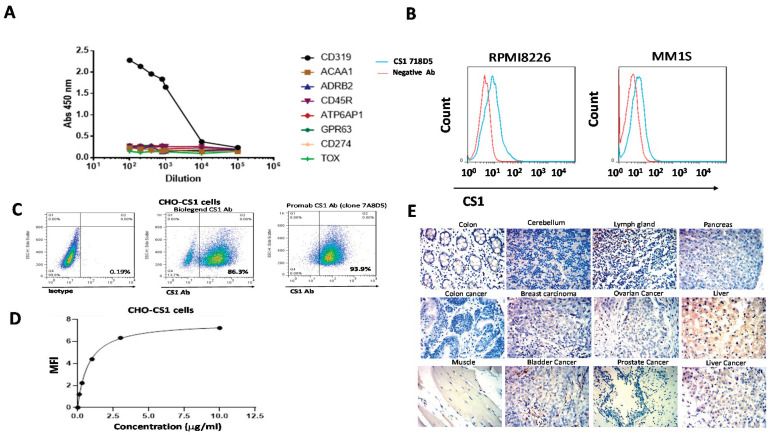
The CS1 (clone 7A8D5) antibody binds the CS1 protein. (**A**) ELISA shows binding to the CS1 antigen but not negative control proteins by the anti-CS1 antibody clone 7A8D5. (**B**) The CS1 antibody binds multiple myeloma cells by FACS. (**C**) The CS1 antibody detects CS1 in CHO-CS1 stable cell line. The commercial CS1 antibody control also bound CS1 (*Biolegend* Clone 162.1). (**D**) Binding affinity by titration of the CS1 antibody using CHO-CS1 cells. The representative curve is shown. The Kd of binding was equal to 0.8 ug/mL or 5.3 nM. (**E**) IHC staining with the CS1 antibody showed positive staining in lymph gland and negative staining in most normal and tumor tissues.

We developed hybridoma clones against the extracellular domain of CS1 and selected the best clone that specifically bound to CS1 antigen. ELISA showed strong and specific binding of the CS1 antibody (clone 7A8D5) to the CS1 extracellular domain protein but not to negative control proteins (Figure 1A). FACS staining detected binding of the CS1 antibody to the extracellular CS1 protein in multiple myeloma cell lines RPMI8226 and MM1S (Figure 1B). In addition, the CS1 antibody (clone 7A8D5) detected CS1 in >93% of CHO-CS1 cells, similarly to the control CS1 antibody (*Biolegend* clone 162.1) (Figure 1C). To detect the affinity of CS1 binding to the CS1 antigen, we performed FACS binding titration of CS1 antibody (clone 7A8D5) using CHO-CS1 cells (Figure 1D). The affinity Kd of CS1 antibody binding was 5.3 nM (Figure 1D). Immunohistochemical staining (IHC) showed that the CS1 antibody did not stain normal and tumor samples with the exception of lymph gland tissues and some liver tissues (Figure 1E). Thus, the new isolated CS1 antibody binds to the CS1 antigen with nanomolar range affinity and high specificity, and it can be used for CAR construct design.

### 3.2. CS1 CAR-T Cells Specifically Target CS1-Positive Target Cells In Vitro

**Figure 2:**
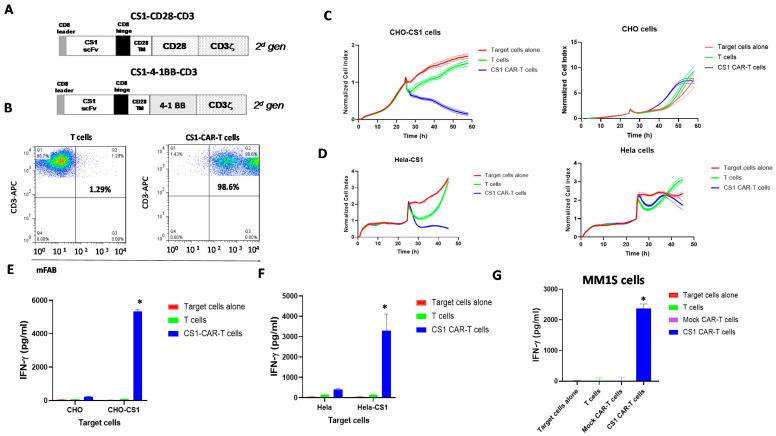
CS1-CAR-T cells target CS1-positive target cells. (**A**) The structure of CS1-CAR constructs. A TM-transmembrane domain, CD28 or 41BB costimulatory domain, and CD3 activation domain were used. (**B**) CS1-CAR-T cells expressed CS1 scFv as detected by FACS with anti-mouse F(ab)’2 antibody. (**C**) CS1-CD28-CD3 CAR-T cells killed CHO-CS1 target cells and did not kill CHO cells. (**D**) CS1-CAR-T cells killed Hela-CS1 and not Hela cells. (**E**) CS1-CAR-T cells secreted higher levels of IFN-gamma than T cells alone with CHO-CS1 cells, * *p* < 0.05, Student’s *t*-test. (**F**) CS1-CAR-T cells secreted significantly higher levels of IFN-gamma with Hela-CS1 cells, * *p* < 0.05 versus T cells. (**G**) CS1-41BB-CD3 CAR-T cells secreted IFN-gamma with multiple myeloma MM1S cells, * *p* < 0.05 versus T cells and Mock CAR-T cells.

We designed lentiviral CS1 clone 7A8D5 ScFv-CAR with either a CD28 or a 41BB costimulatory domain and CD3 activation domain (Figure 2A). After lentiviral transduction, CS1-CD28-CAR-T cells expressed >90% CAR as detected with mouse FAB antibody (Figure 2B). CS1-CD28-CAR-T cells were analyzed using an impedance-based cytotoxicity assay against CHO-CS1 stable cells with CHO as a negative control (Figure 2C). CS1-CAR-T cells specifically targeted CHO-CS1 cells and did not kill CHO cells (Figure 2C). These CAR-T cells also killed Hela-CS1 cells and did not kill Hela cells (Figure 2D). The CAR-T cells secreted a high level of IFN-gamma with CHO-CS1 cells and not with CHO cells (Figure 2E). The same high secretion of IFN-gamma was observed with Hela-CS1 cells but not with Hela cells (Figure 2F). We tested the IFN-gamma secretion of CS1-41BB-CD3 cells with multiple myeloma MM1S cells and found high secretion of IFN-gamma (Figure 2G). Thus, CS1-CAR-T cells specifically targeted CS1-positive target cells.

### 3.3. Bispecific CS1-BCMA CAR-T Cells Target CS1-Positive and BCMA-Positive Target Cells

**Figure 3:**
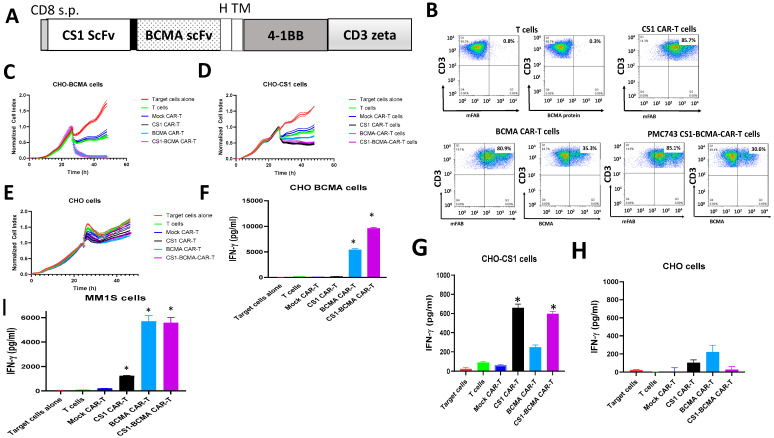
Bispecific CS1-BCMA CAR-T cells specifically target CS1-positive and BCMA-positive target cells. (**A**) Structure of bispecific CS1-BCMA-CAR-T cells. s.p., signaling peptide; H, CD8 hinge; TM-CD28 transmembrane domain; 41BB costimulatory domain; CD3 activation domain. (**B**) Bispecific CS1-BCMA CAR-T cells expressed both ScFvs as detected by FACS with mouse F(ab)2 antibody. BCMA scFv was detected with BCMA recombinant protein. (**C**) Real-time cytotoxicity assay (RTCA) shows activity of bispecific CS-BCMA CAR-T cells with CHO-BCMA target cells. (**D**) RTCA assay shows activity of CS1-BCMA CAR-T cells with CHO-CS1 target cells. (**E**) No cytotoxic activity was with control CHO cells. (**F**) CS1-BCMA-CAR-T cells secreted significantly higher levels of IFN-gamma with CHO-BCMA target cells than Mock CAR-T cells, * *p* < 0.05, Student’s *t*-test, BCMA CAR-T and CS1-BCMA CAR-T cells versus Mock CAR-T cells. (**G**) CS1-BCMA CAR-T cells secreted significantly higher levels of IFN-gamma than Mock CAR-T cells with CHO-CS1 cells, * *p* < 0.05, Student’s *t*-test, CS1-CAR-T cells and CS1-BCMA CAR-T cells versus Mock CAR-T cells. (**H**) A minimal level of IFN-gamma secretion by CS1-BCMA-CAR-T cells was observed with negative control CHO cells. (**I**) A high level of IFN-gamma was detected with CS1-BCMA CAR-T cells co-incubated with target MM1S multiple myeloma cells, * *p* < 0.05 vs. Mock CAR-T cells, Student’s *t*-test, CS1, BCMA and CS1-BCMA CAR-T cells versus Mock CAR-T cells.

To generate bispecific CS1-BCMA CAR, we fused novel CS1 ScFv with BCMA clone 4C8A ScFv described in [29], as well as 41BB costimulatory and CD3 activation domains (Figure 3A). After lentiviral transduction, bispecific CS1-BCMA-CAR-T cells were >80% CAR^+^ by FACS with anti-mouse F(ab)’2 antibody (Figure 3B, lower panel). BCMA Scfv was detected with BCMA recombinant protein (Figure 3B, lower left panels). These CAR-T cells killed both CHO-BCMA (Figure 3C) and CHO-CS1 cells (Figure 3D) and did not kill CHO cells (Figure 3E). CS1-BCMA-CAR-T cells secreted a high level of IFN-gamma against CHO-BCMA cells compared to T and Mock CAR-T cells (Figure 3F). CS1-BCMA CAR-T cells secreted significantly higher levels of IFN-gamma with CHO-CS1 cells than Mock CAR-T cells and T cells (Figure 3G). CS1-BCMA-CAR-T cells secreted minimal levels of IFN-gamma against control CHO cells (Figure 3H). CS1-BCMA-CAR-T cells secreted high levels of IFN-gamma against MM1S multiple myeloma cells (Figure 3I). Thus, bispecific CS1-BCMA-CAR-T cells specifically targeted CS1-positive and BCMA-positive cells in vitro.

### 3.4. G-Rex System-Expanded CS1-BCMA-CAR-T Cells Are Effective against CS1 and BCMA-Positive Target Cells

**Figure 4:**
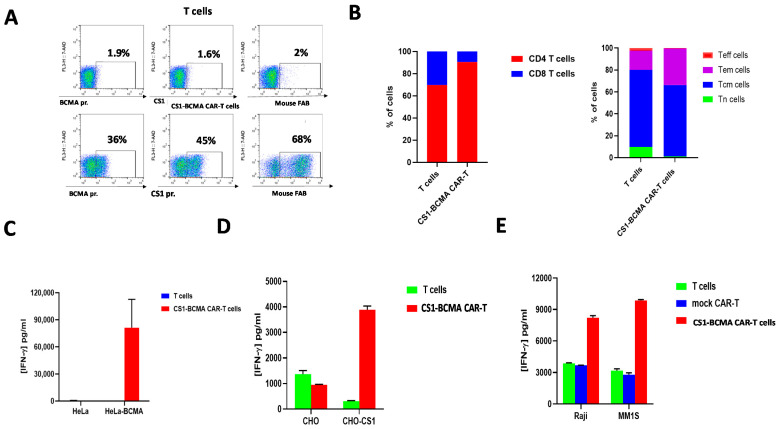
Manufactured with G-Rex system, bispecific CS1-BCMA CAR-T cells express high functional activity with CS1 and BCMA-positive target cells. (**A**) FACS staining detects CS1 scFv and BCMA scFv-positive CAR-T cells. FACS was performed on T cells (top panel) and CS1-BCMA CAR-T cells (bottom panel) with biotinylated recombinant BCMA (left) and CS1 proteins (middle) and mouse F(ab’)2 antibody (right). (**B**,**C**) The percentage of CD4+/CD8+ cells is shown in the left panel, and the percentage of cells with a T_N_, T_CM_, T_EM_, and T_EF_ phenotype is shown in the right panel. (**C**) Manufactured CS1-BCMA CAR-T cells secreted IFN-gamma with Hela-BCMA target cells. (**D**) CS1-BCMA CAR-T cells secreted IFN-gamma with CHO-CS1-positive target cells. (**E**) CS1-BCMA-CAR-T cells had significantly higher levels of IFN-gamma with BCMA/CS1-positive target cells than with T and Mock-CAR-T cells: Raji cells are BCMA-positive cells and MM1S cells are CS1-BCMA-positive cells. (**C**–**E**) *p* < 0.05, Student’s *t*-test, CS1-BCMA CAR-T cells versus Mock CAR-T cells.

Next, we tested the expansion of CS1-BCMA-CAR-T cells in 1 L culture using the G-Rex system for expansion and functional tests in the fresh and frozen state. The CAR-T cells were expanded more than 180-fold during 10 days of expansion resulting in a total of 1.8 × 10^9^ cells. The manufactured CS1-BCMA CAR-T cells were analyzed by flow cytometry for CAR expression, using three biotinylated probes: BCMA protein, CS1 protein, and anti-mouse F(ab’)2 antibody (Figure 4A). FACS staining showed that BCMA protein bound to the BCMA scFv, CS1 protein bound to the CS1 scFv, and anti-F(ab’)2 bound to sequences in both scFvs (Figure 4A). About 30–45% of the cells bound to the BCMA protein and CS1 protein (Figure 4A). The CD4:CD8 ratio of these CAR-T cells was 6:1 (Figure 2B, left panel). The manufactured CS1-BCMA CAR-T cells and control cells were predominantly of a central-memory phenotype (CD62L^+^CD45RA^−^) (Figure 4B, right panel). The CS-BCMA CAR-T cells secreted high levels of IFN-gamma against Hela-BCMA cells (Figure 4C) and CHO-CS1 cells (Figure 4D). We also tested CAR-T cells after freezing and thawing and detected high cytotoxic activity, accompanied by significantly higher levels of IFN-gamma than with T cells against CS1-BCMA-positive MM1S multiple myeloma cells and BCMA-positive Raji cells (Figure 4E). Thus, manufacturing of CAR-T cells using G-Rex system results in high expansion of functional CAR-T cells.

### 3.5. CS1 and CS1-BCMA-CAR-T Cells Effectively Block Multiple Myeloma Tumor Growth and Prolong Mouse Survival

**Figure 5:**
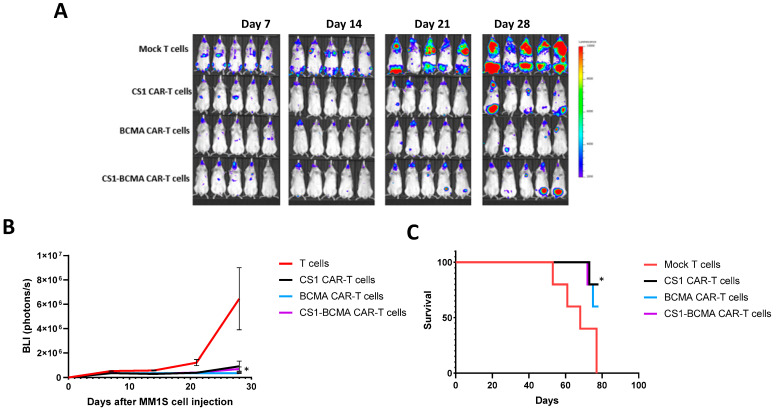
CS1-BCMA CAR-T cells block MM1S multiple myeloma xenograft tumor growth. (**A**) Bioluminescent imaging of mice injected with CS1-CAR-T cells, BCMA-CAR-T cells, CS1-BCMA-CAR-T cells, and mock T cells are shown. (**B**) Quantification of BLI signal. * *p* < 0.05, CS1-BCMA, CS1, and BCMA-CAR-T cells versus T cells, Student’s *t*-test. (**C**) CS1-CAR-T cells and CS1-BCMA CAR-T cells signficiantly prolong mouse survival. Kaplan–Meier curve showing survival of CS1-BCMA CAR-T cells versus control T cells. * *p* < 0.05, ANOVA test.

To test the activity of CS1-CAR-T cells and CS1-BCMA CAR-T cells in vivo, NSG mice were intravenously injected with 2 × 10^6^ MM1S multiple myeloma cells, followed the next day by 1 × 10^7^ frozen T cells or monospecific CS1, monospecific BCMA, or bispecific CS1-BCMA CAR-T cells via the same route. The bispecific CS1-BCMA CAR-T cells significantly decreased xenograft tumor growth (*p* < 0.05 vs. T cells) similar to CS1 or BCMA-CAR-T cells (Figure 5A,B). The CS1 CAR-T, BCMA CAR-T, and bispecific CS1-BCMA-CAR-T cell-treated groups had significantly longer survival versus the Mock control-treated group (Figure 5C). The CAR-T-treated mice did not decrease mouse body weight (not shown). Thus, CS1 and CS1-BCMA-CAR-T cells effectively block multiple myeloma xenograft tumor growth in vivo.

## 4. Discussion

This study demonstrated a novel CS1 antibody specifically recognizing the CS1 antigen with high activity in CAR format. Novel CS1-CAR-T cells effectively targeted CHO-CS1-positive cells and multiple myeloma cells. In addition, we generated novel bispecific CS1-BCMA CAR-T cells and demonstrated high efficacy with both CS1-positive and BCMA-positive cells. We demonstrated that the G-Rex system-manufactured CS1-BCMA-CAR-T cells effectively expanded more than 180-fold with the same functional activity and a high percentage of central memory T cells. In addition, novel CS1-CAR-T cells and CS1-BCMA-CAR-T cells had high in vivo efficacy using the NSG MM1S multiple myeloma xenograft model.

Despite current treatments, multiple myeloma prognosis remains poor. Recently, BCMA-CAR-T cell therapy was approved by the FDA, demonstrating the effectiveness of cellular therapies, but there is still a challenge to overcome patient relapse due to BCMA downregulation. Novel CS1-CAR-T cells and bispecific CAR-T cells targeting both CS1-and BCMA antigens represent one of the approaches to increase the efficacy of CAR-T cells when tumor antigen heterogeneity is present or due to other mechanisms [31]. The bispecific CAR approach used for targeting BCMA and CS1 antigens should be applicable to other MM-associated antigens validated in clinic such as CD38, CD19, and Ig-kappa. The use and combination of different multiple myeloma targets for cellular T therapy will reveal novel signal transduction mechanisms in treating multiple myeloma, clarify resistance to single targeted cell therapy, and provide novel mechanisms of overcoming it by using combination therapy. Different designs, coactivation domains, linkers, hinges, and transmembrane domains can be used for optimizing this bispecific CAR-T cell approach. By combining multiple ScFvs, trispecific CAR-T cells can be also generated in a similar way to CD20-CD19-CD22 CAR-T cells against lymphoma [32]. The use of bispecific and multispecific CAR-T cells is a promising approach to MM and can be extended to solid tumors.

This study demonstrated effective expansion of manufactured CS1-BCMA CAR-T cells using the G-Rex system. The use of the G-Rex system resulted in high expansion of CAR-T cells (>180-fold) with a high number of central memory phenotypes that were shown to be critical for CAR-T cell persistance and increased survival [33,34]. We observed a high percentage of CD4-positive CAR-T cells, suggesting the functional activity of CD4-positive-CAR-T cells [29,35,36].

In summary, this study showed the efficacy and specificity of novel CS1 (7A8D5 Scfv)-CAR-T cells and bispecific CS1-BCMA CAR-T cells, as well as demonstrated the prolonged survival of mice treated with CS1-CAR-T cells and CS1-BCMA-CAR-T cells in an MM1S NSG xenograft model. This study provides a solid basis for future clinical studies of single CS1 and bispecific CS1-BCMA CAR-T cells in the clinic.

## 5. Conclusions

The novel CS1 and bispecific CS1-BCMA CAR-T cells have high efficacy against multiple myeloma in vitro and in vivo. This report provides a basis for future clinical studies.

## 6. Patents

A patent application was filed on the novel CS1 and CS1-BCMA CAR-T cells.

## Data Availability

The data are contained in the article, and all data can be provided.
